# The shaping of onion seedlings performance through substrate formulation and co-inoculation with beneficial microorganism consortia

**DOI:** 10.3389/fpls.2023.1222557

**Published:** 2023-07-12

**Authors:** Robert Pokluda, Lucia Nedorost Ragasová, Miloš Jurica, Andrzej Kalisz, Monika Komorowska, Marcin Niemiec, Gianluca Caruso, Maciej Gąstoł, Agnieszka Sekara

**Affiliations:** ^1^ Department of Vegetable Sciences and Floriculture, Faculty of Horticulture, Mendel University, Brno, Czechia; ^2^ Department of Horticulture, Faculty of Biotechnology and Horticulture, University of Agriculture, Krakow, Poland; ^3^ Department of Agricultural and Environmental Chemistry, Faculty of Agriculture and Economics, University of Agriculture, Krakow, Poland; ^4^ Department of Agricultural Sciences, University of Naples Federico II, Naples, Italy

**Keywords:** *Allium cepa* L., mycorrhiza, *Azospirillum brasilense*, degraded soil, stress biomarkers

## Abstract

**Introduction:**

Smart management in crop cultivation is increasingly supported by application of arbuscular mycorrhizal fungi (AMF) and plant growth-promoting microorganisms (PGPM), which sustain soil fertility and plant performance. The aim of this study was the evaluation of the effects of consortia composed of (*Claroideoglomus claroideum* BEG96, *Claroideoglomus etunicatum* BEG92, *Funneliformis geosporum* BEG199, *Funneliformis mosseae* BEG 95, and *Rhizophagus irregularis* BEG140) and PGPM (*Azospirillum brasilense* – AZ, or *Saccharothrix* sp. – S) on onion cultivated in growing media with a composition corresponding to a degraded soil.

**Methods:**

Three types of substrate formulations were used, with peat:sand ratios of 50:50, 70:30, 100:0 (v:v). The analysis of substrate parameters crucial for its fertility (pH, salinity, sorption complex capacity, and elements’ content) and characteristics reflecting onion seedlings’ performance (fresh weight, stress biomarkers, and elements’ content) was performed.

**Results:**

AMF colonized onion roots in all treatments, showing increasing potential to form intercellular structures in the substrates rich in organic matter. Additionally, co-inoculation with PGPM microorganisms accelerated arbuscular mycorrhiza establishment. Increased antioxidant activity and glutathione peroxidase (GPOX) activity of onion roots sampled from the formulations composed of peat and sand in the ratio of 100:0, inoculated with AMF+S, and positive correlation between GPOX, fresh weight and antioxidant activity of onion roots reflected the successful induction of plant acclimatization response. Total phenols content was the highest in roots and leaves of onion grown in substrates with 70:30 peat:sand ratio, and, in the case of roots, it was correlated with AMF colonization parameters but not with antioxidant activity.

**Discussion:**

AMF and PGPM efficiency in supporting onion growth should be linked to the increased onion root system capacity in mineral salts absorption, resulting in more efficient aboveground biomass production. AMF and PGPM consortia were effective in releasing minerals to soluble fraction in substrates rich in organic matter, making elements available for uptake by onion root system, though this phenomenon depended on the PGPM species. Microorganism consortia enhanced onion seedlings’ performance also in substrates with lower content of organic carbon through plant biofertilization and phytostimulation

## 1 Introduction

Microbial interactions in the rhizosphere are intensified by released plant root exudates, which are the main food source for microorganisms, increasing their population density and activity (Raaijmakers et al., 2009; Gavilanes et al., 2021). Plants benefit from root-associated microorganisms through various activities (phytohormones, nutrient supplementation, and pathogen suppression), ultimately increasing growth, health, and yield, thus decreasing the dependence on harmful chemicals and their after-effects (Raaijmakers et al., 2009; Syed and Tollamadugu, 2019). The potential of microbial symbioses is currently considered a valuable contribution to precision agriculture (Ma, 2019). Such inoculation can increase crop yield by enhancing nutrient uptake and pathogen biocontrol (Parnell et al., 2016; Nacoon et al., 2021). Significant changes in the indigenous microflora of soil by introducing single cultures of exogenous microorganisms appear to be complex, and the efforts have not always been successful (Onishchuk et al., 2017; Iturralde et al., 2019). Therefore, the physiological and ecological compatibility of beneficial and effective microorganisms is an important factor that increases the probability of shifting and controlling the “microbiological equilibrium” of the rhizosphere to encourage the growth, yield, and health of crops (Bidondo et al., 2016).

Onion (*Allium cepa* L.) is an economically important crop that is characterized by high environmental and cultivation demands. Onion seedlings grow relatively slowly and develop shallow weakly branched roots without root hairs, which shows a low efficiency in uptaking soil water and nutrients; thus, they are vulnerable to their deficiency since the beginning of the crop cycle (Sekara et al., 2017; Golubkina et al., 2020). Therefore, it is difficult to supply onion seedlings with sufficient amounts of soil nutrients to ensure optimum growth, especially in the early growth stages (Serra and Currah, 2002). According to Lee and Lee (2014) the optimum ranges of soil parameters should be as follows: pH 6.0–6.5; organic matter 25–35 mg kg^−1^; P 129–168 mg kg^−1^; exchangeable K, Ca, and Mg, 0.39–0.50, 5.8–6.7, and 2.1–2.7 cmol_c_ kg^−1^, respectively. The rate of nutrient uptake depends on the growth stage, as the requirement for N is high during seedling production and subsequent vegetative growth (Mosse et al., 1981; Drost and Koenig, 2002). Simultaneously, high levels of N may cause leaching, denitrification, and increased susceptibility to pests and diseases (Sekara et al., 2017). Arbuscular mycorrhizal fungi (AMF) and plant growth-promoting microorganisms (PGPM) can be successful inoculants when applied at the beginning of the onion vegetation stage to balance the seedling nutritional status (Deressa and Schenk, 2008; Colo et al., 2014). However, the potential benefits justify the investigation of the mechanisms of interactions between AMF, PGPM, and onion plants (Lone et al., 2015; Rozpadek et al., 2016). The research undertaken so far covers the selection of the most effective microorganisms to establish successful symbiose/mutualism in particular environmental conditions and farming systems (Bolandnazar, 2009; Galván et al., 2009; Albrechtová et al., 2012; Caruso et al., 2018), and the implications of these ecological relationships on onion plant performance, especially with respect to bulb yield and quality (Mollavali et al., 2015; Shinde and Shinde, 2016; Fredotovic and Puizina, 2019; Petrovic et al., 2020).

Among the AMF associated with onion roots in different environments and cultivation systems, *Claroideoglomus* spp., *Funneliformis* spp., and *Rhizophagus* spp. are the most widespread (Charron et al., 2001; Bolandnazar et al., 2007; Bolandnazar, 2009; Galván et al., 2009; Mohamed et al., 2014; Mollavali et al., 2015). Concerning the plant growth promoting bacteria, the effects of inoculation with *Azotobacter* sp., *Sphingobacterium* sp., and *Burkholderia* sp. were investigated in onion (Tinna et al., 2020). Hong et al. (2019) demonstrated endophytic infection of cyst-like cells after onion inoculation with *Azospirillum brasilense*. Nevertheless, knowledge regarding the relationship between onions and *Azospirillum* spp. is limited.

Less attention has been paid to AMF and PGPM co-inoculation during the initial stages of onion ontogeny, particularly during transplant production in controlled conditions, where all types of substrates can be used, shaping distinct conditions for the establishment of ecological relationships in the rhizosphere (Joe et al., 2012; Colo et al., 2014; Mohamed et al., 2014; Ma, 2019). However, such experiments provide clear insight in soil–plant–microorganisms system and allow the identification of the most beneficial AMF and PGPM consortia for onions in the juvenile growth stage. Moreover, the established symbiosis can be continued under field conditions bringing multiple advantages during the overall growing cycle, including increased plant nutrient uptake, imparted biotic and abiotic stress tolerance, and better bulb quality characteristics (Tinna et al., 2020).

The aim of this study was to evaluate the effect of onion inoculation with AMF + *A. brasilense* or *Saccharothrix* sp. consortia on onion seedling development in relation to the substrate formulation. The influence of AMF and PGPM consortia on the growing medium was assessed with respect to the parameters crucial for fertility (pH, salinity, sorption complex capacity, and C, N, P, K, Mg, Na, and Ca concentrations) and onion seedling biochemical characteristics reflecting plant performance (fresh weight, stress biomarkers, and concentration of K, P, Mg, Na, and Ca).

## 2 Materials and methods

### 2.1 Material and experimental protocol

The onion (*Allium cepa* L.) cultivar ‘Stalagmit’ F_1_ (Moravoseed, Ltd, CZ) was used for this research. The experiment consisted of seven treatments, each with three replicates (eight plants per replicate). The experimental treatments included three non-inoculated substrate formulations (control) and four substrate formulations inoculated with consortia of arbuscular mycorrhizal fungi (AMF) and plant growth-promoting microorganisms (PGPM). The treatments and abbreviations used in this study are listed in **Table 1**.

**Table 1:** Treatments and their abbreviations used in this study.

Abbreviation	Peat:sand (v:v) ratio	Inoculation
**C 50**	50:50	–
**AMF + AZ 50**	50:50	arbuscular mycorrhizal fungi mix (AMF) + *Azospirillum brasilense* (AZ)
**C 70**	70:30	–
**AMF + AZ 70**	70:30	AMF + AZ
**C 100**	100:0	–
**AMF + AZ 100**	100:0	AMF + AZ
**AMF +S – 100**	100:0	AMF + *Saccharothrix* sp. ST2020 (S)

Sowing peat (Klasmann, DE) and sand (local sources) were used. Calcium carbonate in remarkable amounts was used to maintain a pH of approximately 6.5. The remaining substrate parameters are shown in **Supplementary Table 1**. Before sowing, the substrates were autoclaved at 120°C for 60 min, and then inoculated with AMF and PGPM, namely *A. brasilense* (Tarrand et al., 1978) (CCM 3862) (Czech Collection of Microorganisms, Masaryk University, Brno, Czech Republic), or *Saccharothrix* sp. (ST2020) (AMF + S). *Saccharothrix* species and strain details are currently confidential because of the patent pending. AMF mix was composed of *Claroideoglomus claroideum* BEG96, *Claroideoglomus etunicatum* BEG92, *Funneliformis geosporum* BEG199, *Funneliformis mosseae* BEG 95, and *Rhizophagus irregularis* BEG140 (Symbiom Ltd., Lanškroun, Czech Republic). The AMF mix contained 145 spores per gram, and it was applied at a dose of 0.015 g per cm^3^ of substrate. Inoculation with PGPM was performed on onion seeds soaked for 30 min in *A. brasilense* and *Saccharothrix* sp. (ST2020) suspensions, respectively. *A. brasilense* culture were grown for one week on LuriaAgar media at 24°C (HiMedia Laboratories, Mumbai, India) and ST2020 was grown in a yeast-malt extract liquid medium (ISP2) from the International Streptomyces Project (Shirling and Gottlieb, 1966) at 28°C with agitation at 90 rpm for 10 days. Next, both cultures were homogenized using sterile ceramic beads and the concentration of both suspensions was adjusted to 10^8^ CFU/ml in sterile physiological saline (Joe et al., 2012). The substrate parameters selected at the beginning of the experiment are listed in **Supplementary Table 1**.

### 2.2 Cultivation *c*onditions

The seeds were sterilized for 10 min in 0.5% sodium hypochlorite, washed with sterile distilled water, and sown in Teku V9 containers (square, 9 × 9 cm; height, 8 cm; volume, 512 cm^3^) on 2 April 2020. Seedlings were grown in a phytochamber Fytoscope 4400 (PSI, Czech Republic) at a temperature of 20/18°C (day/night), relative air humidity 80%, light intensity 120 µmol m^−2^ s^−1^ at the germination stage; 18/16°C, 70%, 200 µmol m^−2^ s^−1^, respectively, at the beginning of the cotyledon stage, and 21/18°C, 75%, and 200 µmol m^−2^ s^−1^, respectively, after the first leaf stage, with 16 h of daylight. Seedlings were irrigated with a measured volume of tap water. Urea was used for fertilization (5 May, liquid 0.2% solution, 20 cm^3^ per pot in irrigation doses), later the fertilizer YaraTera Kristalon 20 + 5 + 10 + 2 (N, P, K, Mg) Azur was applied each week until 18 June as a 0.1% liquid solution at a dose of 20 cm^3^ per pot.

### 2.3 Substrate sampling and analyses

At the end of the experiment (26 June 2020), samples of homogenized substrate (150 g per treatment) were collected, air-dried, and basic parameters were determined, including pH (H_2_O and KCl) using the potentiometric method, salinity with the conductometric method, and the sorption complex capacity using Kappen’s method. Total N and organic C were determined via elemental analysis using a Vario Max Cube apparatus (Elementar Analysensysteme GmbH, Langenselbold, Germany). The available forms of macroelements (K, P, Mg, Na, and Ca) after extraction with acetic acid were determined by inductively coupled plasma atomic emission spectrometry using a Perkin Elmer Optima 7600 spectrometer (PerkinElmer, US).

### 2.4 Plant material sampling

Onion plants were collected on 26 June 2020, all leaves were cut with scissors, and roots were completely extracted from the substrate and washed with distilled H_2_O. Samples were stored immediately after harvest in a deep freezer (TSE240VGP, Thermo Fisher Scientific, USA) at temperature −80°C until analysis.

### 2.5 Fresh weight

Total leaf and root fresh weights (FWs) per plant were measured using a Sartorius A120S balance (Sartorius AG, Germany).

### 2.6 Staining and microscopy

For colonization analysis, four randomly selected 10 mm long root section per replicate were sampled, fixed in a formaldehyde:ethanol:acetic acid 10%:50%:5% v/v solution (FAA) and stored in the dark at 4°C before staining for microscopy (Schmidt et al., 2008). The roots were then rinsed in distilled H_2_O, cleared in 2% KOH for 1 h at 50°C, and washed in distilled H_2_O (4 × 3 min). Roots were stained in a tube with a mixture consisting of WGA AF 594 conjugate (Invitrogen, USA) (50 μg ml^−1^), concanavalin A AF 647 (Invitrogen, USA) (50 μg ml^−1^), and acid fuchsine (3%) at a ratio of 1:1:1 for 4–5 h at room temperature, rinsed in 1×PBS (4 × 3 min), and incubated for 12 h in 1×PBS to remove all excess stain. Before mounting on the slide, a few drops of Hoechst stain were added to the slides with the roots (Vierheilig et al., 2005). Mycorrhizal colonization was quantification by evaluating four root segments per replication for mycelia, vesicles, arbuscules, and spore presence. Ten observations were made at each root segment, and the calculation of colonization was based on the ratio of segments with AMF structures to roots without fungal structures (Alarcón and Cuenca, 2005).

Confocal microscopy was completed using the LSM 800 (Carl Zeiss, Germany) microscope at 590/617 nm excitation/emission for WGA AF 594, 650/668 nm for concanavalin A AF 647, and 350/461 nm for Hoechst stain. The lens used was a ×20/0.8 NA. Images were processed using the Zen Blue software (Carl Zeiss, Germany).

### 2.7 Analyses of stress biomarkers

The antioxidant activity against 2,2-diphenyl-1-picrylhydrazyl (DPPH radical) was measured in onion root and leaf samples. For the extraction, 2.5 g of homogenized onion roots or leaves per repetition was weighed, ground with 10 ml 80% methanol, and centrifuged (3,492×*g*, 10 min, 4°C). The mixture containing 0.1 cm^3^ supernatant and 4.9 cm^3^ 0.1 mM DPPH in 80% methanol was incubated in darkness at 20–22°C after 15 min, the absorbance was measured at λ = 517 nm using a UV–VIS Helios Beta spectrophotometer (Thermo Fisher Scientific, Inc., US). Antioxidant activity was calculated using the following formula: DPPH (%) = ((A0 − A1)/A0) × 100, where A0 is the absorbance of the reference solution and A1 is the absorbance of the test solution (Molyneux, 2004).

Total phenol content was determined using the modified Folin–Ciocalteu colorimetric method (Djeridane et al., 2006). For the extraction, a 2.5 g sample of plant material was ground, mixed with 10 cm^3^ of 80% methanol, and centrifuged (3,492×*g*, 15 min, 4°C). The glass tubes were filled with 0.1 cm^3^ of the supernatant and 2 cm^3^ of sodium carbonate, left for 5 min, and then 0.1 cm^3^ of Folin–Ciocalteu’s reagent mixed with deionized water (1:1 v/v) was added. After 45 min, phenols were determined by the colorimetric method at 750 nm using a UV–VIS spectrophotometer against a reference solution. The total phenol value was expressed as gallic acid equivalents (mg GAE) per gram of FW.

Glutathione peroxidase activity (GPOX) was measured according to Lück (1962). Plant samples (2.5 g) were ground in an ice bath with 20 cm^3^ of a 0.05 M potassium phosphate buffer and centrifuged (3,492×*g*, 15 min, 4°C). The reaction mixture contained diluted supernatant, 0.05 M potassium phosphate buffer, p-phenylenediamine, and hydrogen peroxide. The absorbance at 485 nm was recorded at 60 s intervals for 2 min using a UV–VIS spectrophotometer. GPOX activity is expressed as units (U) per g FW per min.

### 2.8 Element concentration in plant tissues

To determine the element concentrations in onion roots and shoots, 0.5 g dry weight (DW) of plant material samples were mineralized in a mixture of HNO_3_ and H_2_O_2_ at 1:3 (v:v), then 2 cm^3^ HNO_3_ per 100 cm^3^ distilled water was added. The samples were concentrated 5-fold, and then the concentrations of elements (K, P, Mg, Na, and Ca) were determined by atomic emission spectroscopy (ICP) with an Optima 7600 spectrophotometer (Perkin Elmer, US) using the method described by Pasławski and Migaszewski (2006).

### 2.9 Statistical analysis

The substrate and plant samples were analyzed for every treatment in three technical replicates, each consisting of eight plants. The data were tested for normality of distribution according to the Shapiro–Wilk method and homogeneity of variances using the Levene test. The ANOVA was applied to test significance levels at *p ≤*0.05 (*), *p ≤*0.01 (**), or *p ≤*0.001 (***) and non-significant (ns), followed by Tukey’s honest significant difference (HSD) test to separate means into homogenous groups. The results were also examined using Pearson’s correlation coefficient (r) between the analyzed parameters. Principal component analysis (PCA) and cluster analysis (CA) were performed to precisely demonstrate and analyze the data and their relationships. Correlations and PCA were used as supplementary statistical methods that enabled and expanded the analysis of the presented data and made additional relationships visible between the experimental treatments and variables. The results using raw data are presented for PCA because no substantial differences appeared between the raw and standardized data. Modeling was performed with “general regression models (GRM)”, a stepwise regression procedure with backward elimination was performed to remove the least significant variables. Multiple regression screening, based on the investigated variables, was performed to determine the most accurate compatibility. Coefficients of determination (R2) and standard errors of estimation (SEe) were calculated to assess the accuracy of the models. All analyses were performed using Statistica 13.3 (Dell, Inc., USA).

We used multiple linear regression analysis to develop models that describe how the y-variable (FW of leaves, roots, and leaves + roots of onion seedlings) is related to several explanatory variables xn (substrate and plant parameters). After stepwise regression analysis with backward elimination of all investigated parameters variables, did not allow to formulate clear model, so variables affecting simultaneously the FW were screened and two models were developed. The first model for onion FW focused on whole-seedling FW and had the following form: FW of roots + leaves = 1.120 ∗ Mg in substrate − 0.004 ∗ Na in the substrate. The input data were the mean content of elements in the substrate and mycorrhizal parameters. The coefficient of determination is high (R2 = 0.968). This means that the regression line approximates the real data points quite well and that the model can explain approximately 97% of the onion seedling DW variation with *p ≤*0.05.

The second model was revealed for onion root FW, with input data including only the colonization rate, arbuscule abundance, and vesicle abundance:


Roots FW=1.18+0.102∗colonization rate−0.0917∗arbuscule abundance−0.046∗vesicles abundance

The coefficient of determination is high (R2 = 0.944). with *p ≤*0.05. According to multiple regression models, the total onion seedling FW can be predicted based on the Mg and Na content in the substrate, whereas root FW can be predicted based on the analyzed AMF parameters.

## 3 Results

### 3.1 Fresh weight of onion as affected by AMF and PGPM application to investigated growing media

**Figure 1:**
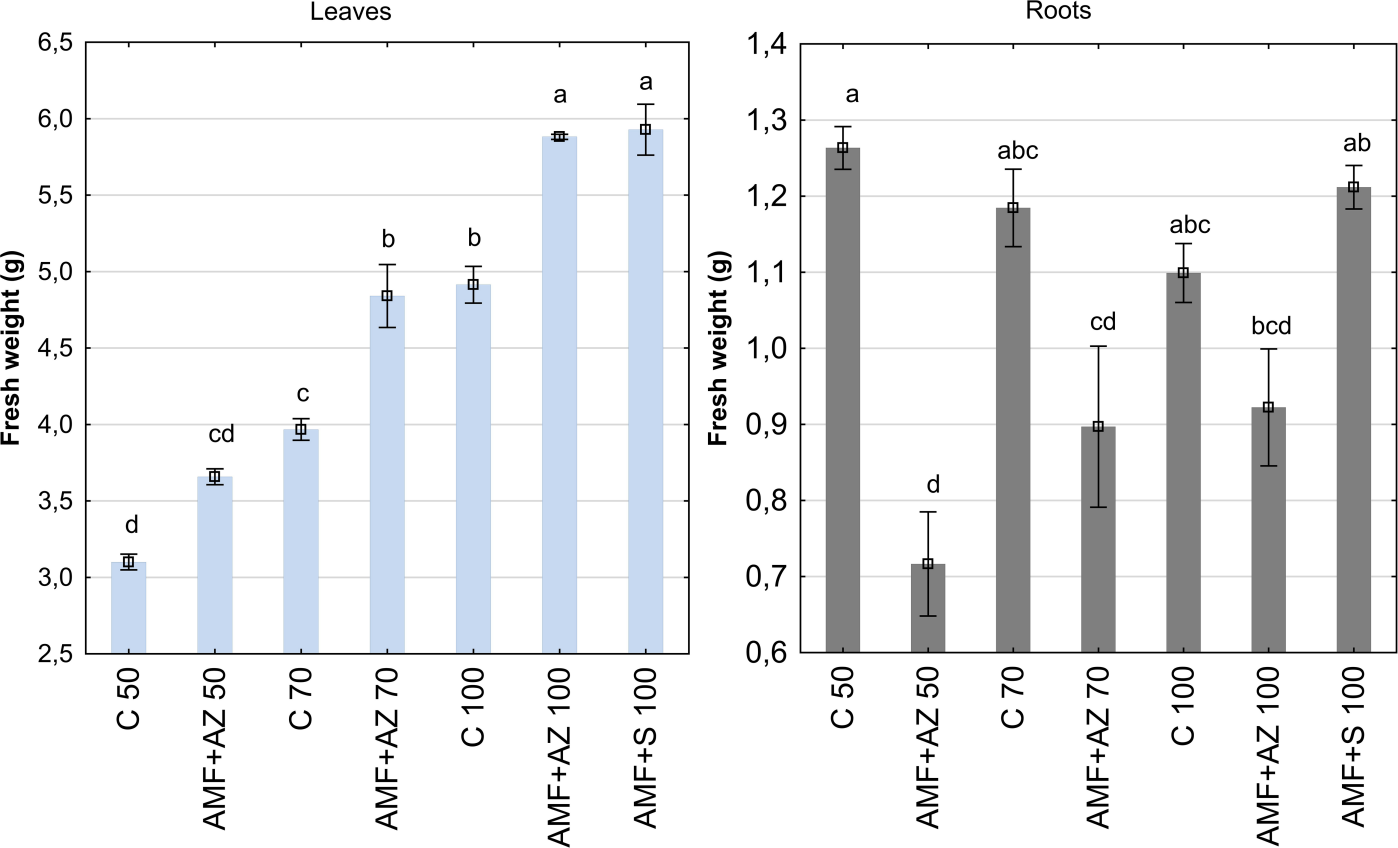
Mean values (± SE) of the fresh weight of onion leaves (left) and roots (right). Bars marked with different letters represent different values at *p ≤*0.05, according to Tukey’s test. C 50, peat:sand ratio 50:50 (v:v) without inoculation; AMF + AZ 50, peat:sand ratio 50:50 (v:v) inoculated with arbuscular mycorrhizal fungi (AMF) and *Azospirillum brasilense* (AZ); C 70, peat:sand ratio 70:30 (v:v) without inoculation; AMF + AZ 70, peat:sand ratio 70:30 (v:v) inoculated with AMF and AZ; C 100, peat:sand ratio 100:0 (v:v) without inoculation; AMF + AZ 100, peat:sand ratio 100:0 (v:v) inoculated with AMF and AZ; AMF + S 100 peat:sand ratio 100:0 (v:v) inoculated with AMF and ST2020 (S).

The fresh weight (FW) of onion leaves and roots at harvest was 10.94 g and 8.54 g on average, respectively, and exhibited significant dependence on the experimental treatments and plant tissues (**Figure 1**; **Supplementary Table 2**). The lowest root FW value was recorded in the AMF + AZ 50 treatment (0.72 g) and the decrease in root FW was 43.3% compared to the control C 50 treatment (1.26 g). The roots of onions sampled from all treatments inoculated with PGPM (AMF + AZ 70, AMF + AZ 100, and AMF + S 100) developed similar root FW as the non-inoculated control with corresponding peat:sand formulations (C 70 and C 100). The FW of onion seedling leaves grown on all substrates inoculated with AMF and PGPM (AMF + AZ 50, AMF + AZ 70, AMF + AZ 100, and AMF + S 100) were significantly higher than in the corresponding controls, where similar peat:sand ratios were used (C 50, C 70, and C 100, respectively). The differences in FW accounted for 18.0% for the AMF + AZ 50 treatment, 22.0% for AMF + AZ 70, 19.7% for AMF + Z 100, and 20.6% for AMF + S 100. Analysis of the total onion seedling FW (leaf + root) revealed that the highest values were recorded for AMF + AZ 100 and AMF + S 100 treatments, 3.40 and 3.57 g, respectively). Onion root FW was negatively correlated with Ca and Mg content in the leaves (**Supplementary Table 3**). Leaf FW was positively correlated with Ca and Mg content in roots, but negatively correlated with Na content in roots. Moreover, leaf FW was positively affected by Ca, Mg, Na, K, and P content in the substrate, AMF colonization rate, arbuscule, and vesicle abundance in the roots.

### 3.2 Root colonization of onion as affected by AMF and PGPM application to investigated growing media

**Figure 2:**
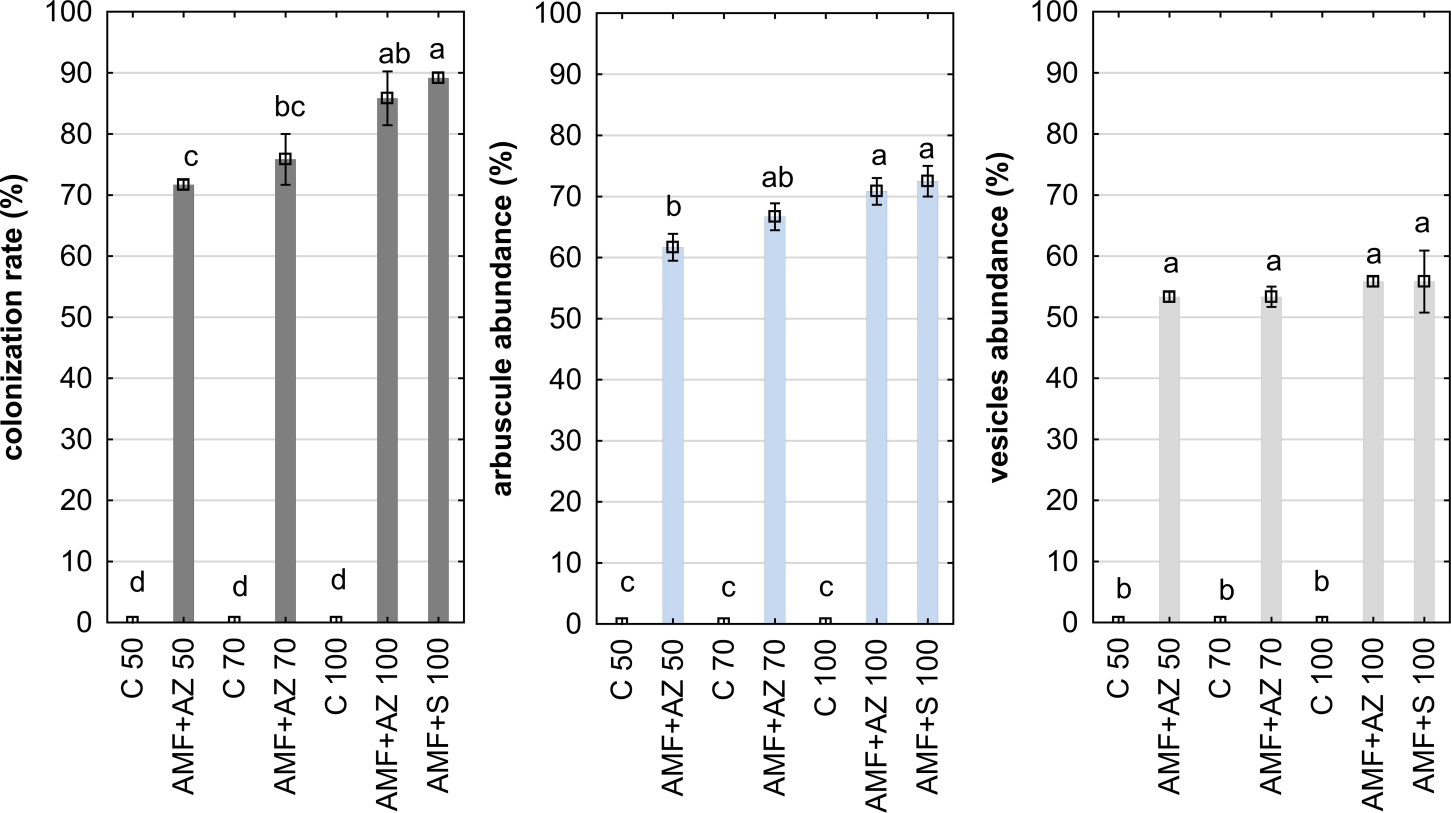
Mean values ( ± SE) of the colonization rate (left), arbuscule abundance (center), and vesicle abundance (right). Bars marked with different letters represent different values at *p ≤*0.05, according to Tukey’s test. Abbreviations are explained in **Figure 1** and **Table 1**.

**Figure 3:**
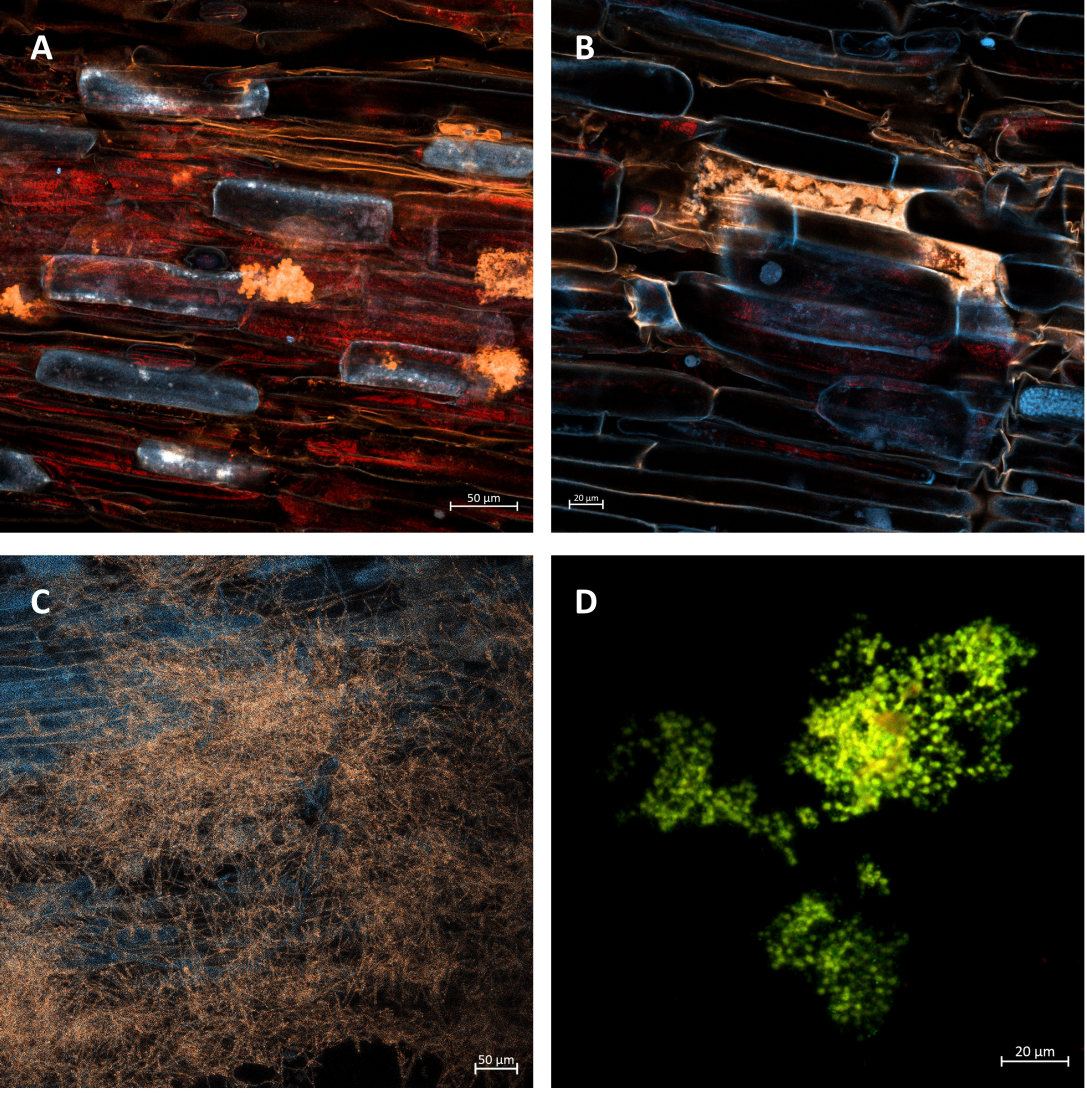
**(A)** Arbuscules (in orange color) developed within the tissue of onion root. Treatment 50 AMF + AZ. Bar = 50 µm. **(B)** Details of arbusculum in particular root cells (orange structure), treated with 100 AMF + AZ. Bar = 20 µm. **(C)** Grid of mycorrhizal mycelia surrounding the onion roots treated with 50 AMF + AZ. Bar = 50 µm. **(D)** Colonies of *Azospirillum brasilense* in the root-hair area. Treatment with 100 AMF + AZ. Scale bar = 20 µm.

The data in **Figure 2** show a high level of root colonization rate in the AMF + AZ 100 and AMF + S 100 treatments (88.3 and 90.0%, respectively). Results of observations performed with confocal microscopy showed successful root colonization of AMF in all inoculated treatments (**Figure 3**). No colonization was observed in the control treatment.

The arbuscules were present in all treatments inoculated with AMF, with the highest value observed in AMF + AZ 100 and AMF + S 100 treatment (73%) (**Figure 2**). Vesicles were observed in onion roots sampled from all inoculated substrate formulations in similar amounts 53%–60% (**Figure 2**). Described parameters indicated the intensive process of mutual symbiosis resulting in the parallel development of AMF, namely colonization rate, arbuscule abundance, and vesicles abundance. This process was additionally confirmed by PCA analysis, showing a close distance between the eigenvectors relevant to colonization rate, arbuscule, and vesicle abundance. Mycorrhization parameters of onion roots (colonization rate, arbuscule abundance, and vesicle abundance) were positively correlated with K content in the substrate. Mycorrhization parameters were positively correlated with antioxidant activity and total phenols in root tissues but negatively correlated with root FW (**Supplementary Table 3**). Concerning leaf tissues, mycorrhization parameters were positively correlated with leaf FW and Ca, Mg, Na, and P.

### 3.3 Analyses of stress biomarkers of onion as affected by AMF and PGPM application to investigated growing media

**Figure 4:**
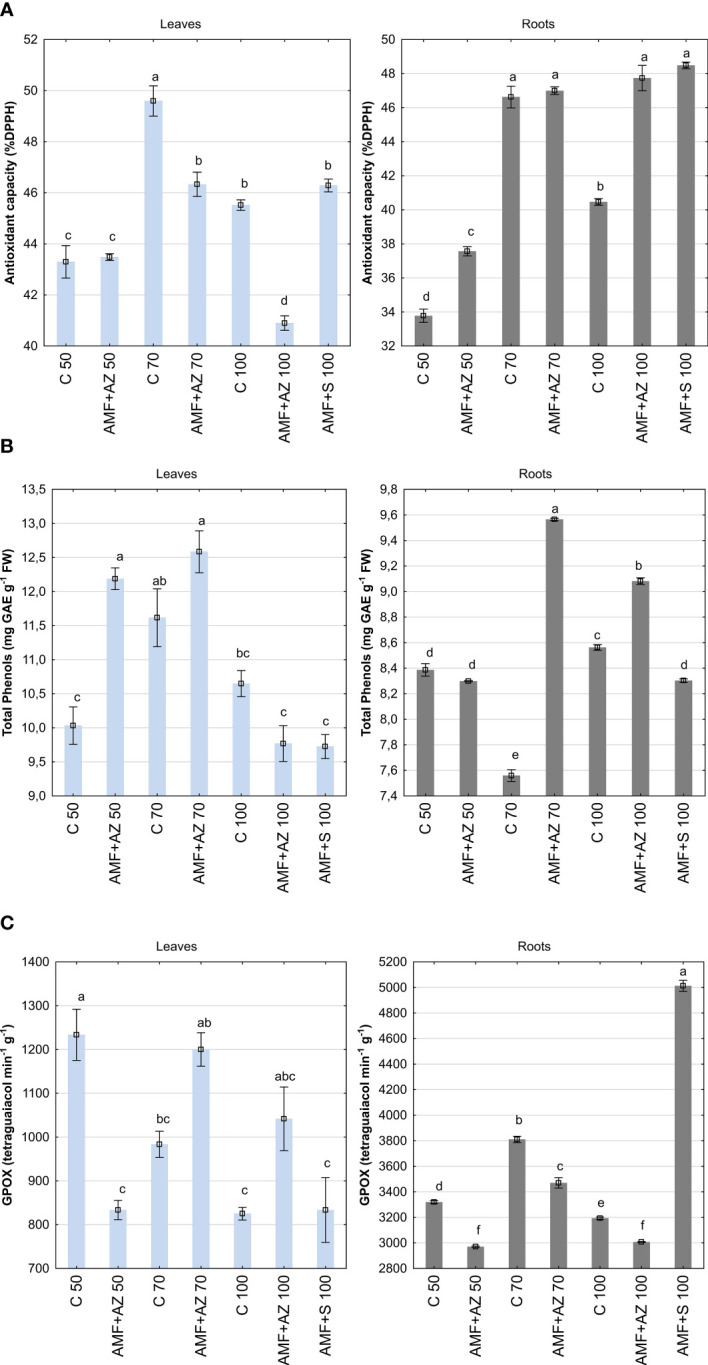
Mean values ( ± SE) of antioxidant activity **(A)**, total phenols **(B)**, and glutathione peroxidase (GPOX) activity **(C)** in the leaves (left) and roots (right) of onion seedlings. Bars marked with different letters are significantly different at *p ≤*0.05, according to Tukey’s test. Abbreviations are explained in **Figure 1** and **Table 1**.

In general, the experimental treatments significantly affected the antioxidant activity of onion seedlings, although no general tendencies were observed. The antioxidant activity, measured as DPPH scavenging activity, was higher in onion seedling roots sampled from the treatments inoculated with AMF and PGPM, namely AMF + AZ 50 (11.2%), AMF + AZ 100 (18.0%), and AMF + S 100 (19.8%), compared to the corresponding controls (C 50 and C 100) at a significance level of 0.05. For onion leaves, the antioxidant activity of plant samples collected from the AMF + AZ 70, AMF + AZ 100 treatments was lower (6.6% and 17.5%, respectively, at a significance level of 0.05) than that of the corresponding controls (C 70 and C 100) (**Figure 4A**; **Supplementary Table 2**). The antioxidant activity of onion root extracts was positively correlated with K content in the substrate, AMF colonization rate, arbuscule and vesicle abundance, and root K, Ca, Mg, and GPOX activity (**Supplementary Table 3**). The antioxidant activity of onion leaf extracts was not affected by soil characteristics or AMF symbiosis, but it was positively correlated with K, Mg, and P content in roots, as well as with GPOX activity in root tissues.

The total phenol content was 35.7% higher in onion seedling leaves than in roots based on the main effect analysis (**Figure 4B**; **Supplementary Table 2**). Onion roots sampled from plants inoculated with AMF + AZ 70 accumulated the highest number of phenolic compounds (9.56 g), 26.5% higher than the corresponding uninoculated control (C 70). In onion leaves, the highest number of total phenols was determined in AMF + AZ 50 and AMF + AZ 70 treatments (12.19 and 12.58 r, respectively), but only in the case of AMF + AZ 50, the mentioned compounds were accumulated at 21.5% higher extent than in plant leaves of corresponding control (C 50). Total phenols of onion root extracts were positively correlated with Ca content in roots, as well as K and Na contents in leaves (**Supplementary Table 3**). A negative correlation was noted between the total phenol content and all elements analyzed in the substrate.

**Figure 5:**
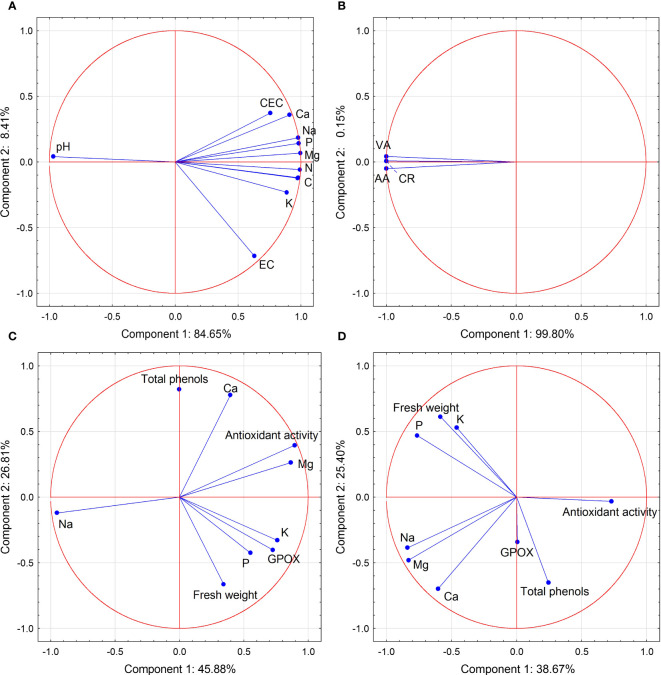
Biplot of PCA of the feature space built using data on the biochemical characteristics of the substrate after cultivation **(A)**, root colonization **(B)**, onion roots **(C)**, and leaves **(D)**. Circles represent observations in the principal component space, whereas vectors indicate the contributions of each feature to the first (x axis) and second (y axis) PCs. EC, cation exchange capacity; EC, electrical conductivity; VA, vesicle abundance; AA, arbuscule abundance; CR, colonization rate; GPOX, glutathione peroxidase activity.

The onion seedling roots showed three-fold higher glutathione peroxidase (GPOX) activity than the leaves, concerning the main effects (**Figure 4C**; **Supplementary Table 2**). The highest GPOX activity was noted in root samples from the AMF + S 100 treatment (3,470 µmol tetraguaiacol min^−1^ g^−1^). The GPOX activity in roots sampled from inoculated AMF + AZ 50 and AMF + AZ 70 treatments was lower (by 5% and 8%, respectively) than that in samples from the corresponding non-inoculated controls (C 50 and C 70). The lowest GPOX activity was noted in onion leaf tissues sampled from treatments with addition of inoculants, namely AMF + AZ 50 and AMF + S 100 (833.3 and 833.8 µmol tetraguaiacol min^−1^ g^−1^, respectively) as well as from non-inoculated C 100 treatment (825.0 µmol tetraguaiacol min^−1^ g^−1^). The GPOX activity in onion leaves of AMF + AZ 70 and AMF + AZ 100 treatments was higher (by 22.0% and 26.3%, respectively) than that recorded in plant samples from the corresponding controls (C 70 and C 100). The GPOX activity of onion root extracts was positively correlated with Mg and negatively correlated with Na content in the roots (**Supplementary Table 3**). No positive correlations were observed between GPOX activity in onion leaf tissues and other soil or plant characteristics. The correlations between the investigated stress biomarkers and the other onion seedling root and leaf characteristics are illustrated in **Figures 5C, D** by the magnitude of the angles between the relevant eigenvectors.

### 3.4 Element’s concentration in onion tissues as affected by AMF and PGPM application to investigated growing media

The mineral content of onion seedlings was significantly affected by substrate composition and microbial inoculation. K and Ca accumulated in higher amounts in onion seedling leaves than in roots. In contrast, Mg, Na, and P contents were higher in the onion seedling roots than in the leaves (**Table 2**).

**Table 2:** Effects of soil microbial inoculants on mineral content in onion seedling roots and leaves (mg kg^−1^ DW) (mean values ± SD).

Treatment	Ca	Mg	Na	K	P
Roots					
C 50*	5,624 ± 301 b **	3,462 ± 20 d	36,255 ± 224 ab	13,640 ± 14 d	4,354 ± 23 ab
AMF + AZ 50	6,530 ± 97 b	5,352 ± 61 c	37,240 ± 561 ab	14,319 ± 10 cd	3,142 ± 18 b
C 70	6,642 ± 417 b	7,162 ± 283 ab	29,522 ± 351 cd	17,571 ± 312 a	4,475 ± 198 a
AMF + AZ 70	10,927 ± 121 ab	7,785 ± 777 a	28,832 ± 1,968 cd	15,046 ± 417 bc	4,537 ± 259 a
C 100	7,649 ± 206 b	6,636 ± 46 abc	38,285 ± 297 a	15,811 ± 244 b	4,285 ± 54 ab
AMF + AZ 100	16,648 ± 560 a	6,190 ± 818 bc	31,671 ± 4,806 bc	14,954 ± 509 bc	3,759 ± 1038 ab
AMF + S 100	9,981 ± 337 b	7,487 ± 695 ab	25,450 ± 1,823 d	15,923 ± 688 b	4,278 ± 444 ab
Leaves					
C 50	11,356 ± 99 c	3,082 ± 2 b	12,956 ± 214 a	29,185 ± 395 b	2,439 ± 78 c
AMF + AZ 50	14,675 ± 1,444 a	3,996 ± 279 a	13,074 ± 49 a	26,718 ± 738 c	2,843 ± 205 b
C 70	11,355 ± 905 c	3,088 ± 217 b	9,319 ± 210 b	28,941 ± 6 b	2,566 ± 16 bc
AMF + AZ 70	13,448 ± 408 ab	3,687 ± 52 a	12,409 ± 342 a	30,398 ± 320 ab	2,571 ± 93 bc
C 100	8,045 ± 348 d	2,498 ± 32 c	9,714 ± 107 b	29,226 ± 954 b	2,838 ± 189 b
AMF + AZ 100	12,624 ± 217 bc	4,000 ± 50 a	13,518 ± 654 a	31,780 ± 395 a	3,345 ± 60 a
AMF + S 100	13,656 ± 305 ab	3,718 ± 69 a	12,722 ± 440 a	29,674 ± 597 b	2,908 ± 100 b

*Abbreviations are explained in **Figure 1** and **Table 1**.

**Values in each column followed by different letters are significantly different at p ≤0.05, according to Tukey’s test.

The calcium content in the onion leaves and roots ranged from 5,624 to 16,648 mg kg^−1^ DW (**Table 2**; **Supplementary Table 4**). Under the conditions of the peat content in the substrate at the level of 50%, the Ca content in the roots was 5,624 mg kg^−1^ DW, while when cultivated on pure peat, this value increased to 7,649 mg kg^−1^ DW. In onion roots cultivated in substrates with inoculation (AMF + AZ 70 and AMF + AZ 100), a significant increase in Ca content was observed in comparison to the corresponding controls (C 70 and C 100). Concerning the Ca content in onion leaves, a significant increase was found in plant material collected from the AMF-S 100 treatment compared to the C 100 treatment.

The Mg content in the tested plants ranged from 2,498 to 7,785 mg kg^−1^ DW (**Table 2**; **Supplementary Table 4**). A significant increase in the concentration of Mg in the onion root tissues by 25%, and in the leaf tissues by 15% was observed as a result of inoculation. The roots collected from the inoculated AMF + AZ 70 and AMF + S 100 treatments contained the highest amount of Mg, but these values were not significantly different from those of the corresponding controls (C 70 and C 100). In the case of onion leaves, inoculation significantly increased Mg content in the AMF + AZ 100 and AMF + S 100 treatments compared to that in the corresponding control (C 100).

In general, approximately 30% more sodium was observed in plants inoculated with substrates than in the corresponding control without mycorrhiza. The level of Na determined in onion roots of the inoculated AMF + AZ 100 and AMF + S 100 treatments was significantly lower than that in the plant material from the corresponding control (C 100). In the case of Na content in onion seedling leaves, no significant differences were noted for the treatments.

There was no difference in the K content of the biomass of plants grown on substrates with and without mycorrhiza. There was also no effect of increasing the proportion of peat in the substrate on the shaping of K content, which ranged from 13,626 to 32,175 mg kg^−1^ DW. Onion roots sampled from the AMF + AZ 70 treatment contained lower amounts of K than the corresponding control (C 70). Similarly, onion leaves of AMF + AZ 70 accumulated less K than those of the corresponding control (C 70); however, in the case of AMF + AZ 100, the dependence was reversed. In the case of K content in onion seedling leaves, no significant differences were noted between the treatments.

The phosphorus content in plant roots ranged from 2,721 to 4,796 mg kg^−1^ DW, whereas that in leaves ranged from 2,360 to 3,405 mg kg^−1^ DW. It was found that the P content was approximately 10% higher inplants collected from substrates subjected to inoculation. P accumulation in onion roots sampled from the inoculated AMF + AZ 50 treatment was lower than that in the samples from the corresponding control (C 50). No significant differences were recorded for the treatments concerning P content in the onion seedling leaves.

The analysis of correlation coefficients indicated a positive relationship between Ca content in the growing medium and P content in the leaves (**Supplementary Table 3**). Ca content in onion roots was positively correlated with Mg, Na, K, and P in the growing medium and with Mg, K, and P content in leaves.

### 3.5 Substrate properties after finishing of the growing cycle

The sum of alkaline and acid cations in the sorption complex resulted from the share of the organic fraction in the substrate formulations, and was the highest in the C 100, AMF + AZ 100, and AMF + S 100 treatments, with an organic carbon content of approximately 50%. Total nitrogen was determined in the range of 4.5%–5.0%, and electrical conductivity (EC) 1.38–2.15 mS (**Table 3**). Organic C was positively correlated with the sum of alkaline and acid cations in the sorption complex and with each of the investigated cations (N, Ca, Mg, Na, K, and P) (**Supplementary Table 5**).

**Table 3:** Effects of soil microbial inoculants on substrate physical and chemical characteristics after onion seedling cultivation (mean ± SD).

Treatment		C 50*	C 70	C 100	AMF + AZ 50	AMF + AZ 70	AMF + AZ 100	AMF + S 100
**Sum in the sorption complex (mMol Na+ kg^−1^)**	alkaline cations (S)	430 ± 24 c**	566 ± 31 b	1,287 ± 56 a	420 ± 42 c	551 ± 57 b	1,198 ± 66 a	1,212 ± 47 c
	acid cations (H)	42 ± 3 c	61 ± 5 b	154 ± 14 a	55 ± 7 bc	54 ± 4 bc	148 ± 9 a	135 ± 16 a
**S + H (mMol kg^−1^)**		472 ± 38 c	627 ± 52 b	1,441 ± 76 a	475 ± 54 c	605 ± 38 b	1,346 ± 69 a	1,347 ± 96 a
**Cation exchange capacity with alkaline cations (%)**		88.5 ± 9.5 a	89.5 ± 7.4 a	92.4 ± 6.7 a	83.4 ± 7.2 a	87.1 ± 5.9 a	91.4 ± 6.6 a	88.5 ± 4.7 a
**Organic carbon (%)**		2.55 ± 0.25 c	7.25 ± 0.32 b	48.16 ± 3.63 a	3.02 ± 0.41 c	7.85 ± 0.52 b	50.25 ± 2.32 a	51.14 ± 6.1 a
**Total nitrogen (%)**		0.305 ± 0.0251 c	0.610 ± 0.0712 b	4.542 ± 0.315 a	0.312 ± 0.034 c	0.635 ± 0.070 b	4.789 ± 0.366 a	4.991 ± 0.509 a
**EC (mS)**		0.45 ± 0.031 d	1.51 ± 0.092 b	1.38 ± 0.142 b	0.89 ± 0.102 c	1.58 ± 0.135 b	1.67 ± 0.184 b	2.15 ± 0.182 a
**pH**	H_2_O	7.33 ± 0.52 a	7.18 ± 0.61 a	6.15 ± 0.46 b	7.12 ± 0.42 a	6.89 ± 0.74 a	6.35 ± 0.59 b	6.37 ± 0.76 b
	KCl	7.11 ± 0.29 a	6.75 ± 0.37 b	5.90 ± 0.32 c	6.88 ± 0.41 ab	6.71 ± 0.23 a	6.21 ± 0.41 c	6.24 ± 0.63 c

*Abbreviations are explained in **Figure 1**.

**Values in each column followed by different letters are significantly different at *p* ≤0.05 according to Tukey’s test.

The contents of Ca, Mg, Na, K, and P forms available to plants in the growing medium analyzed after cultivation were the highest in the formulations composed of peat:sand at a ratio of 100:0 (v:v), namely C 100, AMF + AZ 100, and AMF + S 100 (**Table 3**). Potassium content in the substrates after plant cultivation ranged from 21 to 147 mg kg^−1^ DW. Moreover, substrates composed of peat:sand in the ratio of 100:0 (v:v), with the addition of beneficial microorganisms (AMF + AZ 100 and AMF + S 100), contained the highest level of K after cultivation. In the case of treatments composed of peat:sand at a ratio of 70:30 (v:v), higher contents of Ca (9.6%), Mg (30.4%), Na (15.1%), and K (53.2%) were determined in growing media inoculated with PGPM (AMF + AZ 70), compared to the non-inoculated control (C 70), whereas P content was similar in the corresponding inoculated and non-inoculated treatments.

A statistically significant correlation was observed between the contents of all examined elements in their available forms in the media. The most common was a negative correlation between the pH measured in KCl and the sum of cations in the sorption complex, as well as the content of Ca, Mg, Na, K, and P forms available to plants (**Table 4**). These dependencies are illustrated in **Figure 5A** by the wide angles between the eigenvectors relevant to pH_KCl_ and other soil characteristics.

**Table 4:** Effects of soil microbial inoculants on soluble minerals (mg kg^−1^ DW) in the substrate after onion seedling cultivation (mean ± SD).

Treatment	Ca	Mg	Na	K	P
C 50*	2360 ± 121.1 de**	262.2 ± 2.49 e	211.8 ± 9.46 e	21.65 ± 0.531 f	9.35 ± 0.396 e
AMF + AZ50	2219 ± 34.1 de	252.4 ± 2.50 e	219.9 ± 2.48 e	35.30 ± 1.968 e	11.27 ± 0.284 e
C 70	2187 ± 61.4 e	301.6 ± 1.14 d	267.6 ± 2.08 de	33.95 ± 1.535 e	14.87 ± 0.504 d
AMF + AZ70	2397 ± 70.3 d	393.4 ± 4.37 c	307.9 ± 7.56 d	52.01 ± 1.529 d	16.53 ± 1.342 d
C 100	3213 ± 59.4 a	991.0 ± 7.53 a	1500.4 ± 45.50 a	86.21 ± 1.639 c	72.89 ± 1.949 a
AMF + AZ100	2807 ± 47.7 b	976.1 ± 18.02 a	1350.9 ± 38.09 b	150.66 ± 1.529 b	59.31 ± 2.028 b
AMF + S 100	2620 ± 25.4 c	745.8 ± 10.82 b	863.9 ± 8.08 c	101.13 ± 1.724 a	45.99 ± 1.153 c

*Abbreviations are explained in **Figure 1** and **Table 1**.

**Values in each column followed by different letters are significantly different at p ≤0.05 according to Tukey’s test.

### 3.6 PCA and cluster analyses

The PCA biplot (**Figure 5**) shows the contributions of each determined parameter to the first and second PCs separately for soil characteristics, AMF colonization, and onion seedling root and leaf parameters. The data revealed that PC 1 and PC 2 for substrate characteristics accounted for 93.06% of the total variance within the data set, and pH_KCl_ spread below 0 for PC 1, while the remaining factors contributed positively to this component (**Figure 5A**). All AMF colonization parameters contributed negatively to PC 1 (99.80% of total variance) (**Figure 5B**). PCA analysis of onion root characteristics showed that all variables, except Na and total phenols, had positive input to PC 1 (84.65% of total variance), while total phenols, antioxidant activity, Ca, and Mg content had positive output to PC 2 (26.81% of total variance) (**Figure 5C**). In the case of onion leaf characteristics, antioxidant activity and total phenols had a positive input to PC 1, although its share of total variance was only 38.54%. FW, P, and K contents brought positive load to PC 2 (**Figure 5D**).

**Figure 6:**
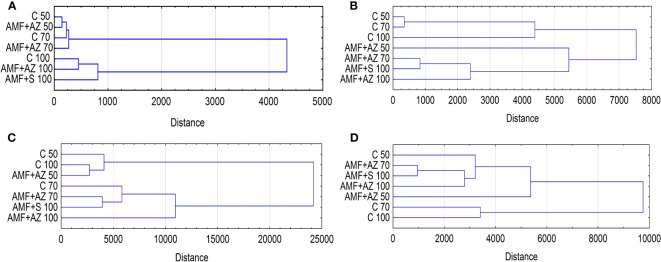
Dendrogram (Euclidean distance, Ward’s method) showing the extent of distance between experimental treatments based on biochemical characteristics of soil after cultivation **(A)**, root colonization **(B)**, onion root **(C)**, and leaf characteristics **(D)**. Abbreviations are explained in **Figure 1** and **Table 1**.

In the dendrogram presented in **Figure 6**, the y-axis shows the distances between the treatments whereas the x-axis represents the analyzed treatments. Based on the cluster analysis (CA) method applied, two main clusters were distinguished based on the substrate characteristics (**Figure 6A**): the first cluster was formed by substrates with a peat:sand ratio of 50:50 and 70:30 (v:v), and the second was formed with a substrate composed of a peat:sand ratio of 100 (v:v). The CA was obtained from the experimental design. CA analysis of root colonization parameters also distinguished two main clusters: the first was composed of non-inoculated treatments (C 50, C 70, and C 100), and the second was inoculated substrate formulations, with the closest distance between the AMF + AZ 70 and AMF + S 100 treatments (**Figure 6B**). The root characteristics of the CA also form two main clusters. The first included the C 50, C 100, and AMF + AZ 50 formulations, and the second included the remaining treatments. CA covering leaf characteristics resulted in grouping of the investigated substrate formulations into clusters separating C 50 and C 70 from the other treatments, while the closest relationship was recorded for AMF + AZ 70 and AMF + S 100 treatments. In general, the fact that certain objects belong to clusters demonstrates their considerable similarity within the group in question.

## 4 Discussion

### 4.1 Fresh weight of onion as affected by AMF and PGPM application to investigated growing media

In the present experiment, the roots of onions from all treatments inoculated with AMF and PGPM had FW similar to that of the uninoculated control treatments in every peat:sand combination. However, the leaf FW of onion seedlings grown on all inoculated substrates was significantly higher than that in the corresponding controls. Albrechtová et al. (2012) confirmed the synergism between AMF and saprotrophic fungi, resulting in a two-fold increase in the onion yield in the presence of organic matter. The mechanism behind the positive effect of inoculation on onion FW can be the synergistic action of AMF, which induces an accumulation of secondary metabolites, vitamins, and minerals (Baslam et al., 2013), and bacteria, such as N-fixing *A. brasilense*, which significantly enhances the length of root hairs and seedling weight by increasing the availability of minerals (Ribaudo et al., 2006). Crops inoculated with *Azospirillum* spp. experience changes in root morphology *via* siderophore production which regulates plant growth as well as vitamins such as thiamine and riboflavin (Sahoo et al., 2014a; Sahoo et al., 2014b). The application of PGPM in vegetables caused a yield increase of above ground crops by 8%–21%, and underground plant parts by approximately 25%–50%, as well as improved nutrient use efficiency by 12%–36% of N, 18%–29% of P, and 9%–15% of K (Rai et al., 2016). The present study confirmed that leaf FW was positively affected by substrate mineral composition, AMF colonization rate, and arbuscule abundance in onion roots. Onion seedling FW was linked to better efficiency but not development of the root system, resulting in higher aboveground biomass production after AMF + PGPM inoculation. Moreover, total seedling biomass can be predicted with the proposed regression equations, including Mg and Na content in the substrate, and root fresh weight can be predicted using regression equations, including AMF parameters (colonization rate, arbuscule abundance, and vesicle abundance).

### 4.2 Root colonization of onion as affected by AMF and PGPM application to investigated growing media

The results of observations performed with confocal microscopy showed successful root colonization of AMF in onion seedlings in all treatments. However, the colonization rate, arbuscule abundance, and vesicle abundance were the highest in substrate formulations based on the peat medium. Paranavithana et al. (2020) confirmed that AMF colonization of rice roots is more effective in soils rich in organic carbon. The described parameters indicated the intensive process of mutual symbiosis resulting in the parallel development by AMF mentioned morphological structures, i.e., arbuscules, that the primary sites of nutrient interchange between roots and fungi (Akiyama and Hayashi, 2006). Additionally, the bacterial strains used in the inocula formulation could accelerate mycorrhiza establishment and development because of their ability to decompose chitin and chitosan, the two main constituents of AMF spore walls, thus supporting spore germination in the onion rhizosphere (Battini et al., 2016). Although *Saccharothrix* spp. produce dithiolopyrolone derivatives with antifungal activity (Merrouche et al., 2017), including PGPM in the formulation used in the present study, the presence of antagonism with AMF was not confirmed. In contrast, such consortium application resulted in improved activity of the enzyme, which is related to lignin formation. Cluster analysis revealed a close distance between AMF + AZ 70 and AMF + S 100 treatments with respect to onion root and leaf characteristics; therefore, AMF and *Saccharothrix* sp. The consortium was efficient in substrates rich in organic C, while AMF and *Azotobacter* sp. could be applied to substrates with lower organic C content. According to a study by Zhou et al. (2020), AMF increase plant carbon sequestration. The combination of AMF and *Saccharothrix* sp. was more effective in organic carbon sequestration and substrate colonization, although other substrate characteristics discussed in the previous subchapter should also be considered. For example, the highest soluble K content in the substrate of AMF + S 100 treatments was analyzed after the experiment. Moreover, the very high antioxidant activity and GPOX activity in onion roots sampled from the AMF + S 100 treatment indicated the successful induction of plant acclimatization, probably linked to *Saccharothrix* sp. and AMF interaction. In a previous study, Merrouche et al. (2017) reported the antagonism of *Saccharothrix* against fungi (e.g., *Fusarium* spp.) and bacteria. Based on present results, it can be concluded that *Saccharothrix* versus AMF interactions can evolve from parasitism under less optimal conditions to synergism under optimal conditions, in which competition for mineral resources is low or absent.

### 4.3 Stress biomarkers of onion as affected by AMF and PGPM application to investigated growing media

Onion seedlings grown in substrates reflecting degraded soil are under abiotic stress, including nutrient imbalances, low water-holding capacity, high concentrations of toxic heavy metals, such as Mn, Fe, and Al, high salinity, and pH fluctuations (Enkhtuya et al., 2000; Lehman et al., 2015). These factors modify the growth, function, and mineral absorption of the root system. AMF and PGPM can improve soil parameters and even compensate for chemical fertilizers, especially in onions, which are described as a highly mycorrhizal-dependent crop (Bidondo et al., 2016). The present study showed a new aspect of this relationship because onion seedling roots grown in substrates inoculated with AMF + AZ 50, AMF + AZ 100, and AMF + S 100 showed higher antioxidant activity than the corresponding controls. The latter indicates the acceleration of anti-stress processes leading to the synthesis of reactive oxygen species scavengers in onion roots. However, in onion leaves of the AMF + AZ 100, and AMF + S 100 treatments, antioxidant activity was significantly lower in leaves than in roots. Antioxidant potency is a measure of the ability of the plants to reverse the toxicity induced by stress factors (Veskoukis et al., 2019). In light of the present results, reinforcing the antioxidant defense of tissues was sufficiently efficient in onion roots in the AMF + AZ 100, and AMF + S 100 treatments, which did not need to be initiated to a similar degree in the onion leaves. AMF and PGPM seem to be moderators of these processes, acting in the rhizosphere by providing mineral nutrients and expressing a variety of enzymes that contribute to the control of cellular ROS levels, as reported by Pozo and Azcón-Aguilar (2007). AMF enhances the concentration of phenolic compounds in the roots (Lokhandwala et al., 2014). In the present study, onion roots sampled from the AMF + AZ 70 treatment accumulated the highest amount of phenol compounds. In onion leaves, the highest number of total phenols was found in AMF + AZ 50 and AMF + AZ 70. Phenolic compounds can scavenge reactive oxygen, which is why the phenolic profile is commonly correlated with the antioxidant activity of plant tissues (Veskoukis et al., 2019). In the present study, total phenols of onion root extracts were positively correlated with AMF (colonization rate, arbuscule, and vesicle abundance), but negatively correlated with antioxidant activity. This phenomenon can be explained by the activity of AMF and PGPM to transform phenols into simple compounds, which are ecologically active in soils through stabilization of free enzymes, modification of the transport and bioavailability of nutrients, and enhancement of element mineralization and humus formation (Raaijmakers et al., 2009).

Additionally, the antioxidant activity of onions is linked to sulfur derivates other than polyphenols (Fredotovic and Puizina, 2019). Moreover, phenols accelerate nutrient uptake by roots by chelating metallic ions and enhancing active absorption sites and soil porosity (Seneviratne and Jayasinghearachchi, 2003). Indeed, the present data revealed a positive correlation between total phenols in onion roots and Ca content in roots, as between K and Na content in leaves. This phenomenon can be explained using polyphenol compounds by AMF as substrates to modify biochemical conditions in the rhizosphere environment (Stewart and Stewart, 2008; Raaijmakers et al., 2009; Fredotovic and Puizina, 2019).

GPOX catalyzes lignin formation and establishes a structural barrier by producing reactive oxygen and nitrogen species (Marjamaa et al., 2009). In the present study, the highest GPOX activity was recorded in root samples from the AMF + S 100 treatment, and the elevated GPOX activity under AMF colonization indicates alleviated oxidative stress and therefore a higher resistance, as was reported by Domokos et al. (2018) for *Artemisia annua* inoculated with *R. irregularis*. Islam et al. (2015) stated that PGPM activated plant antioxidant defense by regulating the activity of GPOX, as a crucial antioxidant enzyme. The next issue is that the GPOX activity in roots sampled from inoculated treatment AMF + AZ 70 was higher as compared to values determined in samples from corresponding controls. AMF and *Saccharothrix* sp. consortium were more effective in organic carbon sequestration of peat media and, in turn, colonization. Moreover, the high antioxidant activity and GPOX activity in onion roots sampled from the AMF + S 100 treatment indicated the successful induction of plant response to stress factors, probably linked to *Saccharothrix* sp. and AMF interaction. In addition, the co-inoculation of onion seedlings with AMF and *A. brasilense* could cause increased GPOX activity in treatments with a lower amount of organic carbon, reflecting abiotic stress. These results are consistent with those previously reported in other plant species, inoculated separately with AMF (Domokos et al., 2018), *A. brasilense* (Checchio et al., 2021), or *Saccharothrix* spp. (Enebe and Babalola, 2019), but no similar effect has been demonstrated for inoculation with AMF and PGPM consortia.

### 4.4 Element’s concentration in onion Tissues as affected by AMF and PGPM application to investigated growing media

Elemental content in plant products is one of the most important quality parameters (Szeląg-Sikora et al., 2016). The chemical composition of plants determines their nutritional value as well as their suitability for processing or long-term storage (Niemiec et al., 2015). Under the conditions of intensive horticulture, a deficiency of macroelements is very common, which results in a reduction in the yield of plants and deterioration in the usable quality of the crop. Deficiency of macroelements in plants occurs even under conditions of high substrate content (Sikora et al., 2020). The level of bioaccumulation of elements in plants depends not only on their amount in the biotope, but also on their properties (Niemiec et al., 2020a). Increasing the degree of absorption of micronutrients is a strategic aspect of the development of agricultural sciences and production practices (Niemiec et al., 2020b; Seymen et al., 2021). The observed macronutrient contents in the onions of all objects were at the optimal level (Wang et al., 2020; Romo-Péreza et al., 2021); however, samples were taken for analysis in the early stages of development. In the case of macronutrients such as calcium or phosphorus, deficiencies are often visible in the later stages of vegetative development or even in the stage of generative development. An increase in the accumulation of macronutrients in young plants reduces the risk of deficiency at harvest maturity (Bana et al., 2022). Research has demonstrated that co-inoculants of AMF and PGPM enhance the nutrient-use efficiency of fertilizers and reduce chemical fertilizer application rates (Bhardwaj et al., 2014). In general, AMF and PGPM directly affect mineral absorption by the host plant by improving plant growth through nutrient acquisition by the fungus, or indirectly by modifying nutrient mobilization from organic substrates, by enhancing fertilizer use efficiency, or by beneficial association with other microorganisms (Vishwakarma et al., 2017). The analysis of available forms of nutrients in the substrate, their uptake by onion roots, and their translocation to shoots was performed in the framework of the present experiment. The onions collected from the substrate with a peat:sand ratio of 100:0, co-inoculated with AMF + AZ, and AMF + S contained the highest levels of Ca and Mg (but lower Na) in leaves. *Azospirillum*-inoculated plants have been reported to cause acidification of the root surroundings, which increases macronutrient and micronutrient uptake (Dobbelaere and Okon, 2007). El-Batanomy (2009) revealed that a mixture of microbial cultures showed the highest nitrogenase activity and mycorrhizal infection in onion roots. The total essential nutrient content in onion dry shoots increased in mixed inocula of *Azospirillum lipoferum*, *A. chrococcoum*, *Bacillus circulans*, *B. polymyxa*, *Rhizobium* sp., and AMF compared to that in the fertilized control. Moreover, Adesemoye et al. (2009) determined another possible mechanism of PRPM and AMF cooperation; namely, the former can act as a vehicle to spread non-mycorrhizal microorganisms throughout the rhizosphere. Bettoni et al. (2014) demonstrated the increase of P level on onion seedlings inoculated with *Glomus intraradices* and organic matter amendment. In the present study, Ca content in onion roots was positively correlated with other elements in the substrate, as well as AMF colonization parameters, antioxidant activity, total phenols in roots, FW of leaves, and K, Mg, and P content in leaves. Moreover, Ca, Mg, K, P, and Na contents in onion leaves were positively correlated with AMF colonization parameters. Surprisingly, relatively few increases in P uptake into the seedlings were observed, which was one of the strongest effects of AMF inoculation. Growing medium composition had a more significant effect on P availability and uptake than AMF-related accumulation of this element by onion seedlings. Mentioned interdependence was reflected in highest soluble P content after cultivation cycle in substrates based on peat (100%) with and without AMF inoculation. This effect is due to competition between the decomposition products of organic matter and P for soil sorption sites, resulting in increased soil solution P concentrations (Guppy et al., 2005). Additionally, the differences between AMF-inoculated and uninoculated plants can be considered the costs paid by plants for AMF-root associations. According to Al-Karakis (2002), AMF inoculation of garlic plants was highest when soil P was lowest, and decreased with increasing P application. Moreover, AMF selectively uptake or make available essential cations to plants, which act as osmotic equivalents for toxic Na ions (Hammer et al., 2011). Moreover, Na ions are compartmentalized in cell vacuoles and mycorrhizal fungal hyphae to avoid translocation to the shoots (Estrada et al., 2013). The interactions between AMF and PGPM concerning mineral salt uptake and synergism can be managed, but one must be aware that synergetic interactions between these microbes could also be manifested.

### 4.5 Substrate characteristics

Onions are among the most sensitive crops to soil conditions, especially salinity, which affects plant growth particularly at the seedling stage (Onishchuk et al., 2017). The physiological and chemical properties of the substrate, including its biomass and chemical composition influence plant growth in a multidirectional manner (Niemiec et al., 2020a). In the present study, the available forms of Ca, Mg, Na, K, and P in the substrate analyzed after cultivation were highest in the formulations composed of peat:sand at a ratio of 100:0 (v:v). Uzinger et al. (2020) demonstrated that the combination of compost and plant growth promoting bacteria resulted in higher P and K availability. In the present study, AMF + PGPM was effective in releasing minerals into the soluble fraction in substrates rich in organic matter, although this capacity was dependent on the consortium of microorganisms used. AMF + AZ 100 and AMF + S 100 application resulted in the highest level of K after cultivation, whereas AMF + AZ 70 inoculants increased the soluble forms of Ca, Mg, Na, and K compared to the non-inoculated control. The P content was similar among the treatments mentioned above. A significant and positive correlation between the soluble elements in substrates and organic carbon, while a negative correlation with pH_KCl_, confirmed the crucial significance of organic matter and reaction for element availability to plants. This phenomenon has been noted by numerous researchers (Guaya et al., 2020; Niemiec et al., 2020b; Lei et al., 2021). Additionally, in the present study, the mineral soluble forms were not reduced in formulations with higher EC values, particularly in the inoculated treatments. Balliu et al. (2015) stated that uptake of elements was enhanced in inoculated tomato seedlings even under moderate salt stress. Despite the large differences in the organic fraction content of the substrates and the capacity of the sorption complex, slight differences in substrate pH occurred after the experiment. In general, a wide spectrum of soil properties converge to create synergistic effects, leading to the reshaping of microbiome/plant interactions.

## 5 Conclusions

Alliaceae crops encounter various challenges, and limiting conditions can result in serious economic losses at every growth stage. Research on plant growth-promoting microbes (PGPM) as a potential component of mainstream agriculture is important and essential. Inoculation with AMF and PGPM consortia resulted in significant improvement in onion seedling performance, i.e., higher aboveground biomass production and better stress adaptation in cultivation in substrates with lower organic carbon content. Our results demonstrated that AMF and *Saccharothrix* sp. were effective in substrates containing 70%–100% peat, while AMF and *Azotobacter* sp. can be recommended to inoculate substrates with 50% organic C to enhance onion seedling performance. Differences between AMF- and PGPM-inoculated plants concerning fresh weight, stress biomarkers, and element concentration were considered the costs paid by plants due to AMF–root associations linked with growing medium-related availability of nutrients. The present findings have revealed a significant aspect of soil/plant management, i.e., that organic/beneficial microbe synergy might be the future key for effective nutrient management and sustainable production. Although we demonstrated the benefits of AMF and PGPM inoculation on onion seedlings, the maintenance of symbiosis after transplanting can be the goal of subsequent investigations, as field conditions create new challenges to plant–microorganism interactions.

## Data availability statement

The raw data supporting the conclusions of this article will be made available by the authors, without undue reservation.

## Author contributions

Conceptualization, RP, AS, AK, and LR. Methodology, RP and LR. Validation, AS and RP. Formal analysis, MN, LR, MJ, and MK. Resources, AS. Data curation, RP and AS. Writing—original draft preparation, RP and AS. Writing—review and editing, LR, MK, MG, MJ, MN, and GC. Supervision, RP. Project administration, AS. Funding acquisition, RP and AS. All authors listed have made a substantial, direct, and intellectual contribution to the work and approved it for publication.
