# Supplementary material for: The shaping of onion seedlings performance through substrate formulation and co-inoculation with beneficial microorganism consortia
Source: Front Plant Sci. 2023 Jul 12;14:1222557. doi: 10.3389/fpls.2023.1222557 (PMC10382143; doi:10.3389/fpls.2023.1222557)
Supplement: Supplementary file 1 [file Table_1.docx]

***Supplementary Material***

**The shaping of onion seedlings performance through substrate formulation and co-inoculation with beneficial microorganism consortia**

Robert Pokluda^1^, Lucia Nedorost Ragasová^1^*, Miloš Jurica^1^, Andrzej Kalisz^2^, Monika Komorowska^3^, Marcin Niemiec^3^, Gianluca Caruso^4^ , Maciej Gąstoł^2^ and Agnieszka Sekara^2^

*** Correspondence:** Lucia Nedorost Ragasová: [lucia.ragasova@mendelu.cz](mailto:lucia.ragasova@mendelu.cz)

**Supplementary tables**

**Table 1.** Selected substrate parameters at the beginning of the experiment (mean values ± SD)

| **Peat: sand treatment** | **pH** | **Dry matter** | **N-NO_3_** | **N-NH_4_** | **N total** | **K** | **P** | **Mg** | **Na** | **Ca** | **Cation exchange capacity** | **Soil weight per pot** | **Substrate bulk density** |
| --- | --- | --- | --- | --- | --- | --- | --- | --- | --- | --- | --- | --- | --- |
|  | CaCl | % | mg kg^-1^ | | | | | | | | mmol kg^-1^ | g | kg m^-3^ |
| 50/50 | 6.53±0.41 a* | 93.0±4.7 b | 5.2±0.4 a | 1.9±0.2 a | 7.0±0.5 a | 505±42 a | 61±6 a | 596±64 a | 92± 8 a | 4435±562 a | 284±22 a | 386± 29 c | 755±52 c |
| 70/30 | 6.64±0.54 a | 90.2±3.1 ab | 6.7±0.3 b | 2.3±0.1 b | 8.9±0.6 b | 684±74 b | 81±5 b | 785±77 b | 113±9 b | 4741±319 ab | 319±37 a | 311±34 b | 606±49 b |
| 100/0 | 6.47±0.43 a | 86.1±2.4 a | 8.9±0.6 c | 2.9±0.2 c | 11.8±0.9 c | 953±69 c | 111±8 c | 1070±112 c | 144±11 c | 5200±461 b | 372±36 b | 163±21 a | 259±31 a |

*Values in a column followed by different letters are different at the level of significance p≤ 0.05 with comparison performed with Tukey test.

**Table 2.** Effects of soil microbial inoculants application to substrates of different organic matter content on fresh weight, antioxidant activity, total phenols and glutathione peroxidase (GPOX) activity of onion seedlings (mean values ± SD)

| **Treatment** | **Fresh weight**  **(mg kg^-1^ DW)** | **Antioxidant activity**  **(%DPPH)** | **Total phenols**  **(mg GAE g^-1^ FW)** | **GPOX**  **(µmol tetraguaiacol min^-1^ g^-1^)** |
| --- | --- | --- | --- | --- |
| C 50* | 2.18±0.01 d** | 38.5±5.28 e | 9.21±0.95 c | 2277±235 b |
| AMF+AZ 50 | 2.58±0.26 c | 48.1±3.26 a | 9.59±2.14 c | 2397±217 b |
| C 70 | 3.01±0.53 b | 43.0±1.88 c | 9.61±2.27 c | 2009±548 b |
| AMF+AZ 70 | 2.19±0.02 d | 40.6±0.68 d | 10.2±1.694 b | 1902±124 c |
| C 100 | 2.87±0.29 c | 46.7±2.79 b | 11.07±1.16 a | 2335±297 b |
| AMF+AZ 100 | 3.40±0.72 a | 44.3±3.85 c | 9.42±0.48 c | 2024±157 c |
| AMF+S 100 | 3.57±0.59 a | 47.4±1.25 a | 9.01±0.82 c | 2923±229 a |

*Abbreviations:

C 50 – peat:sand ratio 50:50 (v:v) without inoculation;

AMF+AZ 50 – peat:sand ratio 50:50 (v:v) inoculated with arbuscular mycorrhizal fungi (AMF) and *Azospirillum brasilense* (AZ);

C 70 – peat:sand ratio 70:30 (v:v) without inoculation;

AMF+AZ 70 – peat:sand ratio 70:30 (v:v) inoculated with AMF and AZ;

C 100 – peat:sand ratio 100:0 (v:v) without inoculation;

AMF+AZ 100 – peat:sand ratio 100:0 (v:v) inoculated with AMF and AZ;

AMF+S – 100 peat:sand ratio 100:0 (v:v) inoculated with AMF and ST2020 (S)

**Values in a column followed by different letters are different at the level of significance p≤ 0.05 with comparison performed with Tukey test.

**Table 3.** Pearson’s correlation coefficients among studied attributes of substrates after cultivation and onion characteristics (N=21)

|  | **FW_R** | **FW_L** | **AAc_R** | **AAc_L** | **TP_R** | **TP_L** | **GPOX_R** | **GOPX_L** | **Ca_R** | **Ca_L** | **Mg_R** | **Mg_L** | **Na_R** | **Na_L** | **K_R** | **K_L** | **P_R** | **P_L** | **Ca_S** | **Mg_S** | **Na_S** | **K_S** | **P_S** | **CR** | **AA** | **VA** |
| --- | --- | --- | --- | --- | --- | --- | --- | --- | --- | --- | --- | --- | --- | --- | --- | --- | --- | --- | --- | --- | --- | --- | --- | --- | --- | --- |
| FW_R | 1 |  |  |  |  |  |  |  |  |  |  |  |  |  |  |  |  |  |  |  |  |  |  |  |  |  |
| FW_L | -0.047ns | 1 |  |  |  |  |  |  |  |  |  |  |  |  |  |  |  |  |  |  |  |  |  |  |  |  |
| AAc_R | -.0123ns | .781*** | 1 |  |  |  |  |  |  |  |  |  |  |  |  |  |  |  |  |  |  |  |  |  |  |  |
| AAc_L | .318ns | -.091ns | 0.315ns | 1 |  |  |  |  |  |  |  |  |  |  |  |  |  |  |  |  |  |  |  |  |  |  |
| TP_R | -.408ns | .402ns | .201ns | -0.468* | 1 |  |  |  |  |  |  |  |  |  |  |  |  |  |  |  |  |  |  |  |  |  |
| TP_L | -.487* | -.351ns | -.010ns | 0.357ns | .120ns | 1 |  |  |  |  |  |  |  |  |  |  |  |  |  |  |  |  |  |  |  |  |
| GPOX_R | .512* | .390ns | .489* | 0.510* | -.297ns | -.287ns | 1 |  |  |  |  |  |  |  |  |  |  |  |  |  |  |  |  |  |  |  |
| GOPX_L | .143ns | -.259ns | -.104ns | -0.143ns | .385ns | .057ns | -.223ns | 1 |  |  |  |  |  |  |  |  |  |  |  |  |  |  |  |  |  |  |
| Ca_R | -.200ns | .714*** | .549** | -0.385ns | .559** | -.248ns | -.045ns | .183ns | 1 |  |  |  |  |  |  |  |  |  |  |  |  |  |  |  |  |  |
| Ca_L | -.503* | .055ns | .218ns | -0.176ns | .170ns | .272ns | .182ns | .049ns | .187ns | 1 |  |  |  |  |  |  |  |  |  |  |  |  |  |  |  |  |
| Mg_R | -.050ns | .618** | .828*** | 0.512* | .169ns | .212ns | .456* | -.284ns | .232ns | .053ns | 1 |  |  |  |  |  |  |  |  |  |  |  |  |  |  |  |
| Mg_L | -.582** | .278ns | .331ns | -0.406ns | .336ns | .125ns | .031ns | .037ns | .490* | .927*** | .072ns | 1 |  |  |  |  |  |  |  |  |  |  |  |  |  |  |
| Na_R | -.079ns | -.627** | -.857*** | -0.4428* | -.086ns | 0.002ns | -.702*** | .148ns | -.242ns | -.319ns | -.748*** | -.288ns | 1 |  |  |  |  |  |  |  |  |  |  |  |  |  |
| Na_L | -,334ns | .113ns | -.088ns | -0.729*** | .483* | -.218ns | -.065ns | .235ns | .308ns | .666** | -.289ns | .735*** | .022ns | 1 |  |  |  |  |  |  |  |  |  |  |  |  |
| K_R | .226ns | .286ns | .615** | 0.753*** | -.454* | .123ns | .432ns | -.398ns | -.064ns | -.231ns | .635ns | -.273ns | -.616** | -.735*** | 1 |  |  |  |  |  |  |  |  |  |  |  |
| K_L | .143ns | .613** | .570** | -0.223ns | .547** | -.419ns | .089ns | .365ns | .660** | -.085ns | .328ns | .121ns | -.406ns | .221ns | .045ns | 1 |  |  |  |  |  |  |  |  |  |  |
| P_R | .401ns | .054ns | .223ns | 0.457* | .037ns | -.028ns | .335ns | .153ns | -.303ns | -.298ns | .279ns | -.429ns | -.519** | -.312ns | .447* | .334ns | 1 |  |  |  |  |  |  |  |  |  |
| P_L | -.268ns | .668** | .393ns | -0.538* | .252ns | -.419ns | -.092ns | -.347ns | .684** | .074ns | .194ns | .392ns | -.100ns | .359ns | -.057ns | .387ns | -.458* | 1 |  |  |  |  |  |  |  |  |
| Ca_S | .094ns | .572** | .125ns | .268ns | .315ns | -.463* | -.075ns | -.291ns | .351ns | -.597** | .163ns | -.368ns | .080ns | -.172ns | .042ns | .423ns | .060ns | .486* | 1 |  |  |  |  |  |  |  |
| Mg_S | .061ns | .803*** | .400ns | .292ns | .306ns | -.557** | .060ns | -.354ns | .565** | -.405ns | .315ns | -.117ns | -.174ns | -.060ns | .167ns | .556* | .014ns | .707*** | .913*** | 1 |  |  |  |  |  |  |
| Na_S | .051ns | .718*** | .300ns | .307ns | .252ns | -.539* | -.037ns | -.386ns | .495* | -.486* | .244ns | -.197ns | -064ns | -.121ns | .157ns | .485* | -.013ns | .686** | .934*** | .989*** | 1 |  |  |  |  |  |
| K_S | -.082ns | .892*** | .580** | .423ns | .389ns | -.548* | .098ns | -.246ns | .785*** | -.043ns | .336ns | .278ns | -.340ns | ,228ns | .098ns | .664** | -.119ns | .856*** | .677** | .900*** | .851*** | 1 |  |  |  |  |
| P_S | .080ns | .732*** | .326ns | .230ns | .218ns | -.529* | .046ns | -.433ns | .462* | -.490* | .297ns | -.226ns | -.106ns | -.165ns | .208ns | .452* | .011ns | .655** | .943*** | .987*** | .992*** | .828*** | 1 |  |  |  |
| CR | -.515* | .607** | .515* | .348ns | .541* | -.030ns | .201ns | -.092ns | .614** | .756*** | .354ns | .876*** | -.458* | .680** | -.212ns | .264ns | -.330ns | .543** | -.050ns | .196ns | .094ns | .504* | .090ns | 1 |  |  |
| AA | -.531* | .596** | .512* | .370ns | .562** | -.046ns | .166ns | -.086ns | .607** | .757*** | .358ns | .882*** | -.452* | .689** | -.219ns | .282ns | -.317ns | .542** | -.051ns | .194ns | .094ns | .515* | .085ns | .9.97*** | 1 |  |
| VA | -.542* | .562** | .474* | .337ns | .511** | -.082ns | .188ns | -.132ns | .557** | .785*** | .329ns | .887*** | -.438* | .677** | -.215ns | .187ns | -.342ns | .509** | -.090ns | .150ns | .052ns | .462* | .050ns | .993*** | .992*** | 1 |

***, ** and *—significant at 0.001, 0.01 and 0.05 levels, respectively; ns—non-significant. FW_R – fresh weight of roots; FW_L - fresh weight of leaves; AAc_R – antioxidant activity of roots; AAc_L – antioxidant activity of leaves; TP_R – total phenols in roots; TP_L - total phenols in leaves; GPOX_R – glutathione peroxidase activity in roots; GOPX_L - glutathione peroxidase activity in leaves; Ca_R – calcium in roots; Ca_L – calcium in leaves; Mg_R – magnesium in roots; Mg_L – magnesium in leaves; Na_R – sodium in roots; Na_L – sodium in leaves; K_R – potassium in roots; K_L – potassium in leaves; P_R – phpsphprus in roots; P_L – phosphorus in leaves; Ca_S – calcium in substrate; Mg_S - magnesium in substrate; Na_S – sodium in substrate; K_S – potassium in substrate; P_S – phosphorus in substrate, CR - colonization rate; VA - vesicels abundance; AA - arbuscule abundance

**Table 4.** Effects of soil microbial inoculants on mineral content in onion seedling roots and leaves (mg kg^-1^ DW)

| **Treatment** | **Ca** | **Mg** | **Na** | **K** | **P** |
| --- | --- | --- | --- | --- | --- |
| C 50 | 8490±3146 c | 3272±208 c | 25620±13874 b | 21412±1144 b | 2992±150 ab |
| AMF+AZ 50 | 10602±4553 bcd | 4674±765 b | 25157±13240 ab | 20510±6807 c | 3397±109 b |
| C 70 | 8998±2657 bc | 5125±2243 ac | 19420±11068 d | 23256±1548 a | 3520±153 ab |
| AMF+AZ 70 | 12187±1406 ab | 5736±2297 a | 20620±9083 cd | 22722±8415 a | 3554±191 ab |
| C 100 | 7847±335 d | 4567±2267 b | 22984±14554 abc | 22518±7374 a | 3561±102 ab |
| AMF+AZ 100 | 14636±4174 a | 5095±1307 ab | 22595±10405 bc | 23367±9224 a | 3552±195 ab |
| AMF+S 100 | 11818±2033 abc | 5736±2110 a | 19086±7071 d | 22799±7554 a | 3593±104 a |

*Abbreviations: see Table 2

**Table 5.** Pearson’s correlation coefficients among studied attributes of substrates after cultivation of onion (N=7)

|  | **S** | **H** | **S+H** | **CEC** | **pH_H2O_** | **pH_HCl_** | **C_org_** | **N** | **K** | **P** | **Mg** | **Na** | **Ca** |
| --- | --- | --- | --- | --- | --- | --- | --- | --- | --- | --- | --- | --- | --- |
| S | 1 |  |  |  |  |  |  |  |  |  |  |  |  |
| H | .991*** | 1 |  |  |  |  |  |  |  |  |  |  |  |
| S+H | .999*** | .993*** | 1 |  |  |  |  |  |  |  |  |  |  |
| CEC | .718ns | .601ns | .715ns | 1 |  |  |  |  |  |  |  |  |  |
| pH_H2O_ | .664ns | .624ns | .659ns | .326ns | 1 |  |  |  |  |  |  |  |  |
| pH_HCl_ | -.969*** | -.966*** | -.969*** | -.619ns | -.666ns | 1 |  |  |  |  |  |  |  |
| C_org_ | .993*** | .986*** | .993*** | .665ns | .670ns | -.957** | 1 |  |  |  |  |  |  |
| N | .990*** | .982*** | .990*** | .653ns | .661ns | -.949** | .999*** | 1 |  |  |  |  |  |
| K | .870** | .887** | .872** | .566ns | .656ns | -.856* | .902*** | .899** | 1 |  |  |  |  |
| P | .967*** | .978*** | .969*** | .752ns | .515ns | -.963*** | .943** | .936** | .830* | 1 |  |  |  |
| Mg | .972*** | .980*** | .973*** | .748ns | .562ns | -.967*** | .961** | .954** | .900** | .988*** | 1 |  |  |
| Na | .947*** | .968*** | .951** | .764* | .466ns | -.942** | .928** | .920** | .852* | .994*** | .987*** | 1 |  |
| Ca | .875** | .980*** | .877** | .749ns | .319ns | -.898** | .836* | .828* | .685ns | .954* | .926** | .949** | 1 |

***, ** and *—significant at 0.001, 0.01 and 0.05 levels, respectively; ns—non-significant. S - sum of alkaline cations in the sorption complex; H – sum of acid cations in the sorption complex; S+H - sum of alkaline and acid cations in the sorption complex; CEC - cation exchange capacity; pHH2O; pHHCl;

Corg – organic carbon; N – nitrogen; K – potassium; P – phosphorus; Mg – magnesium; Na – sodium; Ca - calcium
